# Treatment of beta amyloid 1–42 (Aβ_1–42_)-induced basal forebrain cholinergic damage by a non-classical estrogen signaling activator *in vivo*

**DOI:** 10.1038/srep21101

**Published:** 2016-02-16

**Authors:** Andrea Kwakowsky, Kyoko Potapov, SooHyun Kim, Katie Peppercorn, Warren P. Tate, István M. Ábrahám

**Affiliations:** 1 Centre for Neuroendocrinology and Department of Physiology, Otago Medical School, University of Otago, Dunedin, New Zealand; 2 Centre for Brain Research and Department of Anatomy and Medical Imaging, Faculty of Medical and Health Sciences, University of Auckland, Auckland, New Zealand; 3 Department of Biochemistry, Otago Medical School, University of Otago, Dunedin, New Zealand; 4 MTA-NAP-B-Molecular Neuroendocrinology Research Group, Centre for Neuroscience, Szentágothai Research Centre, Institute of Physiology, Medical School, University of Pécs, Pécs, Hungary

## Abstract

In Alzheimer’s disease (AD), there is a loss in cholinergic innervation targets of basal forebrain which has been implicated in substantial cognitive decline. Amyloid beta peptide (Aβ_1–42_) accumulates in AD that is highly toxic for basal forebrain cholinergic (BFC) neurons. Although the gonadal steroid estradiol is neuroprotective, the administration is associated with risk of off-target effects. Previous findings suggested that non-classical estradiol action on intracellular signaling pathways has ameliorative potential without estrogenic side effects. After Aβ_1–42_ injection into mouse basal forebrain, a single dose of 4-estren-3α, 17β-diol (estren), the non-classical estradiol pathway activator, restored loss of cholinergic cortical projections and also attenuated the Aβ_1–42_-induced learning deficits. Estren rapidly and directly phosphorylates c-AMP-response–element-binding-protein and extracellular-signal-regulated-kinase-1/2 in BFC neurons and restores the cholinergic fibers via estrogen receptor-α. These findings indicated that selective activation of non-classical intracellular estrogen signaling has a potential to treat the damage of cholinergic neurons in AD.

Alzheimer’s disease (AD) is the most common form of dementia and is a progressive neurodegenerative disorder of insidious onset that causes gradual loss of memory and cognitive function^1^. The loss of memory and cognitive function is underpinned by widespread death of neurons in the hippocampus and cortex^1^,^2^,^3^. The basal forebrain cholinergic (BFC) system provides a substantial projection to the hippocampus and cortex to promote cortical arousal, attention and cognitive function and is particularly vulnerable in AD^4^,^5^,^6^. Besides of the pathological aggregation of tau protein^7^ the other major neuropathological hallmark of AD is the accumulation of extracellular neurotoxic amyloid-β (Aβ) peptides such as Aβ_1–42_ in the brain^8^,^9^. Current strategies to combat AD have so far largely been ineffective resulting in an inability to treat the profound cell loss that occurs in AD^1^,^10^,^11^. Among many different factors controlling the vulnerability of cholinergic neurons to AD, estradiol is an essential contributor. Despite the fact that chronic administration of estrogens can improve the synaptic connectivity in the cerebral cortex following neuronal cell death^12^,^13^,^14^ and estradiol was found to be neuroprotective in *in vitro* and *in vivo* models of the disease there is a controversy about estrogen’s neuroprotective actions in AD^15^,^16^,^17^,^18^,^19^,^20^. However, the most common method of chronic administration of estrogens is also associated with detrimental effects, such as an increased risk of stroke and breast cancer^21^,^22^,^23^. One strategy to overcome this shortcoming has been to use synthetic compounds with selective properties of estradiol that do not exhibit negative side-effects during prolonged treatment^21^,^24^. Besides its classical genomic action, estradiol also exerts rapid effects on cells by altering cytoplasmic signal transduction pathways (non-genomic or non-classical for estrogen action)^25^,^26^,^27^ and it is these non-classical actions that are important for mediating the ameliorative effects of estradiol^15^,^28^,^29^,^30^,^31^,^32^. However, estradiol activates both the classical and non-classical pathways and so a selective non-classical estradiol pathway activator is required to induce ameliorative effects without inducing the classical pathway with the risk of unwanted side effects. “Activators of Non-Genomic Estrogen Like Signaling” (ANGELS) such as estren (4-estren-3α, 17β-diol) mimic estradiol’s rapid induction of cell signaling pathways in bone cells, successfully maintaining bone health in gonadectomized mice^33^, while exerting no action on reproductive tissues via classical nuclear receptors^24^. To elucidate the ameliorative effect of ANGELS on the neurodegenerative process we examined the effect of estren on Aβ_1–42_-induced cholinergic damage in BFC *in vivo*.

## Results

### Aβ_1–42_ induces BFC cell body and fiber loss *in vivo*

**Figure 1:**
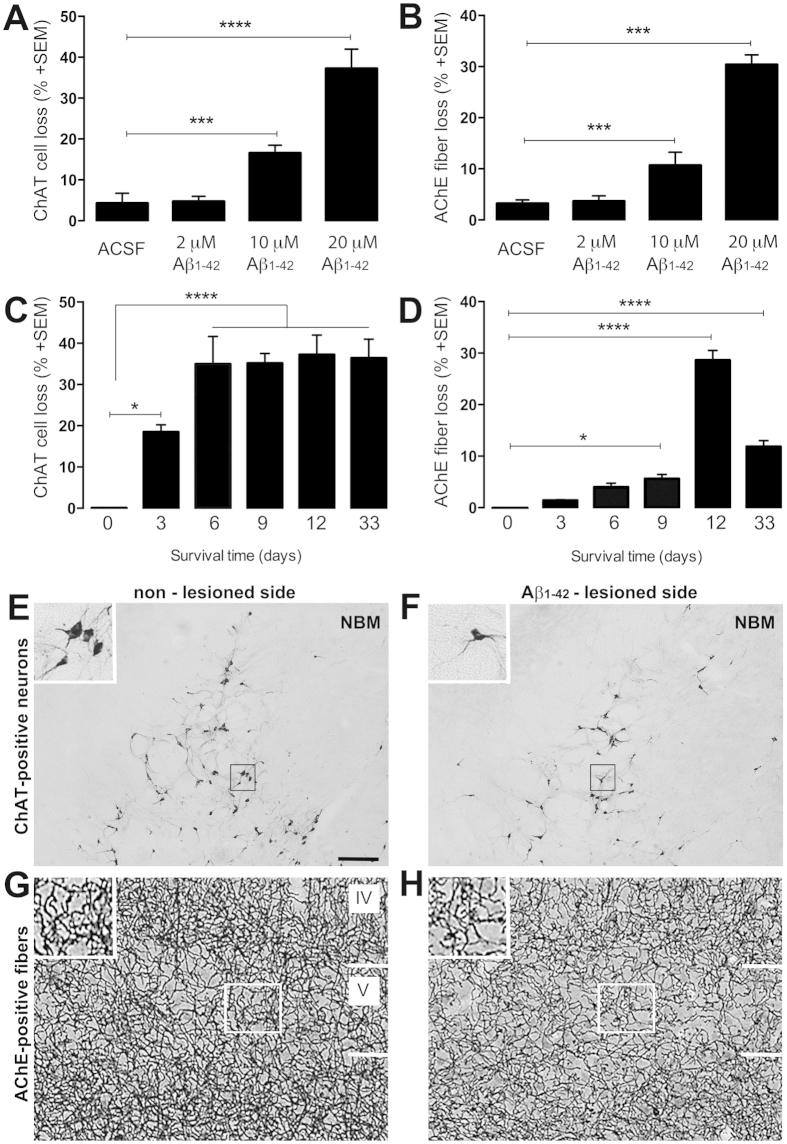
Effect of administering Aβ_1–42_ to NBM on loss of cholinergic cells and fibers. Histogram shows the percentage of ChAT- and AChE-positive cell and fiber loss 12d after administration of different Aβ_1–42_ concentrations (**A**,**B**) and at various survival times after a fixed concentration (20 μM) of Aβ_1–42_ (**C**,**D**). ChAT immunolabeled cell bodies in the NBM (**E**,**F**) and AchE-positive fibers in layer IV and V of the somatosensory cortex (**G**,**H**) at the non-treated (**E**,**G**) and Aβ_1–42_-treated brain side (**F**,**H**). Scale bars, 50 μm; inserts 25 μm. Histograms show mean ± SEM (n = 4–9). ***P < 0.001 (one way ANOVA with post hoc Bonferroni test).

First, we evaluated the most effective dose of Aβ_1–42,_ and survival time of neurons after Aβ_1–42_ application into nucleus basalis magnocellularis (NBM) in ovariectomized (OVX) mice. To facilitate oligomerization and the neurotoxic effect we aged Aβ_1–42_ for 2 days. The most neurotoxic concentration of Aβ_1–42_ was 20 μM (Fig. 1A,B,E,F), and after 12 days (Fig. 1C,D,G,H) the microinjection of Aβ_1–42_ into the NBM complex caused the most profound damage, eliminating 37% of cholinergic cells in NBM and 30% of cholinergic fibers from the somatosensory cortex. Interestingly, Aβ_1–42_-induced cortical cholinergic fiber loss is attenuated by day 33 following Aβ_1–42_ microinjection (Fig. 1D) suggesting an endogenous restoration capacity of remaining cholinergic fibers.

### Single dose of estren treatment restores cholinergic fiber density in the somatosensory cortex after Aβ_1–42_ lesion

**Figure 2:**
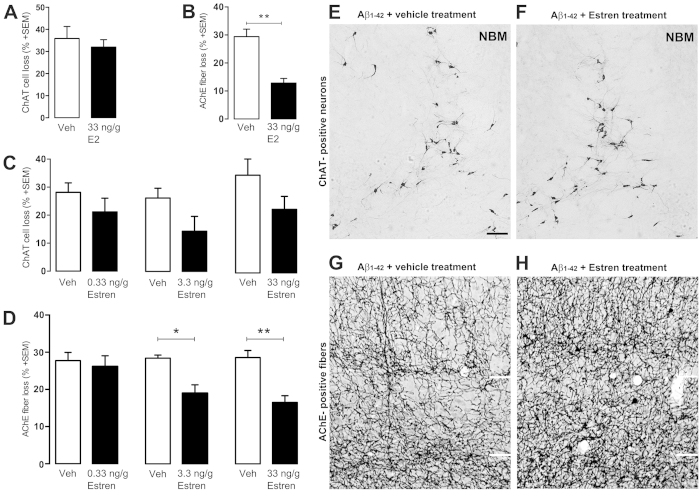
Estren attenuates Aβ_1–42_–induced lesions of cortical projections. Aβ_1–42_ -induced ChAT cell and fiber loss in estradiol (**A**,**B**), estren (**C**–**F**) treated mice compared to the vehicle treated group. Photomicrographs demonstrate ChAT positive cell bodies in the NBM and AChE-stained fibers in layer IV and V of the somatosensory cortex at the contralateral nonlesioned (**E**,**G**) brain side and ipsilateral lesioned (**F**,**H**) side after 12 d of Aβ_1–42_ injection and estren administration. Scale bar, 50 μm. Histograms show mean ± SEM (n = 4–9). *P < 0.05; **P < 0.01; ***P < 0.001 (t-test (**A**,**B**) or ANOVA with post hoc Tukey test).

Next, we tested whether estren has an ameliorative action on Aβ_1–42_-induced cholinergic cell death and fiber loss using different concentrations of estren that do not have uterotrophic effects^24^. We treated the animals with a single dose of estren after Aβ_1–42_ injection. Administration of estren evoked a clear concentration-dependent decrease in cholinergic fiber loss in the lesioned ipsilateral somatosensory cortex with the most significant decrease at 33 ng/g estren treatment (Fig. 2D,G,H) (p < 0.01). In contrast, estren treatment did not have an effect on cholinergic cell loss in the NBM (Fig. 2C,E,F). As a positive control, application of single uterotropic dose of estradiol demonstrated a similar restorative action on BFC fibers but did not have an effect on cholinergic cell loss (Fig. 2A,B).

### Estren ameliorates the learning deficits resulting from Aβ_1–42_ administration

**Figure 3:**
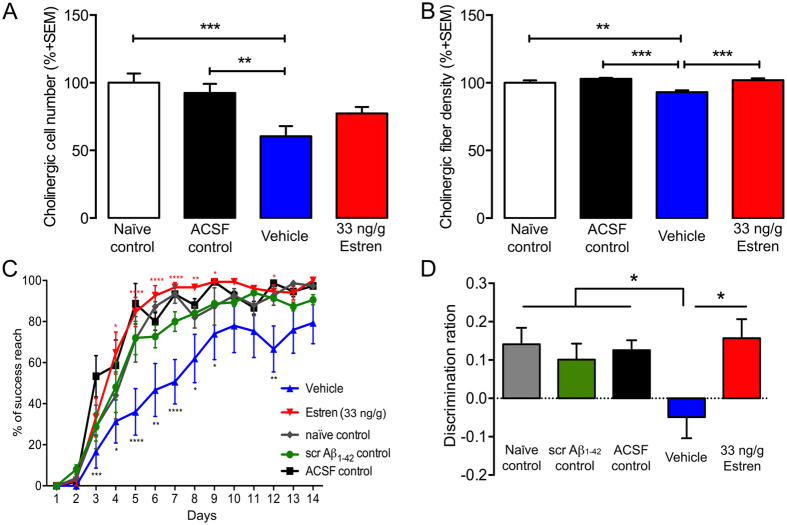
Effect of estren treatment on Aβ_1–42_ -induced behavioural deficits. Aβ_1–42_ -induced bilateral ChAT neuronal (**A**) and fiber (**B**) loss in estren treated mice compared to control groups. Estren treatment successfully rescued reaching performance on the single pellet retrieval task (**C**) and novel object recognition (**D**) in Aβ_1–42_ –lesioned mice. Histograms show mean ± SEM (n = 10). *P < 0.05; **P < 0.01; ***P < 0.001 (ANOVA (**A**) and repeated-measures ANOVA (**B**) with post hoc Bonferroni test).

In the following experiments we examined whether the estren-induced ameliorative effect has a behavioural manifestation. Previous studies have demonstrated that lesions of BFC system are associated with a striking deficit in motor learning and recognition memory detected by pallet retrieval and novel object recognition task, respectively^34^. Accordingly, OVX mice were treated in the same manner as detailed above, with the exception that Aβ_1–42_ or scrambled Aβ_1–42_ was injected bilaterally into the NBM, and then the pellet retrieval and novel object recognition tests were performed 12 d following Aβ_1–42_ or scrambled Aβ_1–42_ application and estren treatment (Fig.3).

Estren significantly attenuated the deficits in successful pellet retrieval, and in the discrimination index in novel object recognition following Aβ_1–42_ administration (Fig. 3C,D). At the end of the behavioral experiments the brains of animals were examined for cholinergic cell and fiber loss (Fig. 3A,B). The behavioural observations in these experiments were supported by the morphological data since estren treatment significantly (p < 0.001) increased the cholinergic fiber density in somatosensory cortex after bilateral Aβ_1–42_-induced cholinotoxicity in NBM (Fig. 3B).

### Estren rapidly and directly activates intracellular signaling system in BFC neuron

**Figure 4:**
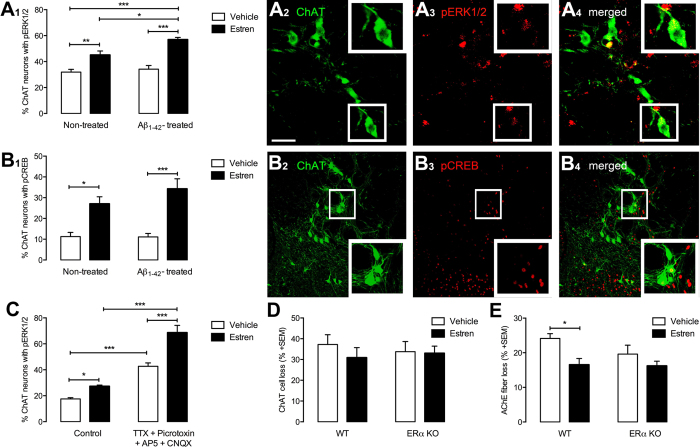
Estren rapidly phosphorylates ERK1/2 and CREB in cholinergic neurons in the NBM. Graphs show the percentage of ERK1/2 (**A1**) and CREB (**B1**) phosphorylation in ChAT neurons within the NBM 30 min after estren administration. Photomicrographs demonstrate dual-label immunofluorescence of pERK1/2 (**A2**–**A4**) or pCREB (**B2**–**B4**) immunoreactivity (red) in ChAT neurons (green). Bar graphs showing the percentage of ChAT neurons expressing pERK1/2 immunoreactivity in the NBM 30 min after incubation *in vitro* in ACSF containing vehicle (open bars) or estren (closed bars) with or without 0.5 μM TTX and amino acid receptor blocker cocktail (10 μM CNQX, 20 μM AP5, 50 μM picrotoxin) (**C**). The effect of estren treatment on cholinergic cells and fibers after Aβ_1–42_ lesion in neuron-specific ERα knock out mice (**D**). Scale bars, 20 μm (A2-A4) and 40 μm (**B2**–**B4**). Histograms show mean ± SEM (n = 5–6). *P < 0.05; **P < 0.01; ***P < 0.001 (ANOVA with post hoc Bonferroni test).

Previous *in vitro* study exhibit clearly how estren attenuates Aβ_1–42_ toxicity in cortical neurons in tissue culture via activation of the mitogen-activated-protein-kinase (MAPK) signaling pathway^35^. We have shown that the non-classical estradiol intracellular signaling pathway such as MAPK/cAMP response element binding protein (CREB) pathway plays a critical role in estradiol-induce ameliorative actions on BFC neurons *in vivo*^32^. To assess whether estren can activate the non-classical pathway in cholinergic neurons in NBM extracellular-signal-regulated-kinase-1/2 (ERK1/2) and CREB phosphorylation was examined in cholinergic neurons in NBM following Aβ_1–42_ and estren administration. While Aβ_1–42_ alone did not change phosphorylation estren increased the ERK1/2 and CREB phosphorylation in cholinergic neurons alone and in Aβ_1–42_ injected animals within 15 minutes (Fig. 4A1). In addition estren-induced ERK1/2 phosphorylation is more prominent in the presence of Aβ_1–42_ (Fig. 4A1). This rapid action of estren on CREB and ERK1/2 phosphorylation suggests a non-classical mechanism that does not require *de novo* gene transcription. Previous studies have revealed that inhibition of either protein synthesis or transcription is ineffective in modulating CREB phosphorylation in such a restricted time frame^36^. We also examined the possible role of afferent inputs to cholinergic neurons in NBM in the rapid estren response by incubating acute brain slices in a cocktail containing TTX and amino acid receptor blockers that effectively isolate cholinergic neurons from synaptic inputs *in vitro*. Our data showed that estren phosphorylated ERK1/2 in the presence of the blocking cocktail, suggesting a GABA_A_/NMDA/AMPA/kainate receptor independent direct estren-induced non-classical action on BFC neurons (Fig. 4C). In these experiments the application of the blocking cocktail alone induced significant increase of ERK1/2 in BFC neurons that might be explained by the pivotal role of amino acid receptors in the regulation of basal ERK1/2 phosphorylation in these neurons.

### Estren restores the cholinergic fiber density via neuronal estrogen receptor α

BFC neurons predominantly express classical estrogen receptor α (ERα)^37^ and our recent data demonstrated the restorative effects of estradiol on BFC neurons are indeed mediated by ERα^32^. We used a neuron-specific ERα KO mouse to examine the role of ERα in estren-induced action on BFC neurons following Aβ_1–42_ toxicity. Our results showed a single dose of estren does not affect Aβ_1–42_-induced cell loss (Fig. 4D) in NBM in the absence of this receptor, but needs ERα to exert a restorative action on cholinergic fiber density (Fig. 4E) suggesting a critical role of neuronal ERα in estren-induced restorative action on BFC neurons in NBM.

## Discussion

We report here that a single dose of estren treatment restores cholinergic fiber density in the somatosensory cortex and it effectively reduces the motor learning and recognition memory deficits after Aβ_1–42_-induced loss of subcortical cholinergic input. This restorative action was absent in neuronal ERα KO mice. Furthermore *in vitro* and *in vivo* experiments demonstrated that BFC neurons in the mouse respond to estren in a rapid and direct manner through an MAPK/CREB signaling pathway.

A feature of the pathogenesis of AD is the increased concentration of toxic soluble oligomers of Aβ peptides^38^. Several laboratories including our own have shown that soluble Aβ oligomer injection into the NBM causes cell loss in NBM, unilateral cortical cholinergic fiber loss in the somatosensory cortex *in vivo* and concomitant memory deficits^39^,^40^,^41^. The available transgenic animal models of AD do not effectively model the sporadic forms of AD and the cholinergic deficit accompanying the disease^42^,^43^,^44^. Based on the established use of soluble Aβ oligomers, we applied the Aβ_1–42_ by intracerebral injection in our experiments and our result demonstrated the Aβ_1–42_ was able to damage one third of the BFC neurons in NBM and impairs learning memory.

Estrogen-induced sprouting effect that is thought to contribute to the neural benefits of estrogen treatment depends upon activation of intracellular signal transduction pathways, including MAPK and CREB^12^,^45^,^46^,^47^,^48^. It is worth noting that the estren treatment we have used here does not exert ERα mediated classical genomic action on uterus^24^. In contrast, estren restores BFC neurons via ERα that does not necessarily involve genomic processes^32^,^35^,^36^. Indeed, estren rapidly increases the number of BFC neurons with phosphorylated MAPK and CREB suggesting that non-classical actions may be involved in estren-induced restorative mechanisms in BFC neurons. Our experimental data also provide evidence that estren directly phosphorylates MAPK and CREB in BFC neurons and restores the cholinergic fibers via ERα. Previous reports, including our own studies, also demonstrate that ERα is highly involved in the estrogen-mediated neurotrophic and sprouting mechanisms in the basal forebrain and in other brain regions^32^,^49^,^50^,^51^.

Estrogen-induced responses in different neuronal cell types range from enhancement of survival, to prevention of cell death and to facilitation of neurite outgrowth^12^. The origin of the estren or estradiol-induced cholinergic fiber restoration is not clear, it is likely that estren or estradiol may enhance the endogenous capacity of the surviving neurons to replace the cortical projections that are lost when BFC neurons begin to die following Aβ_1–42_-induced neurotoxicity^32^. This possibility is supported by the fact that more than 50% of cholinergic neurons survived in NBM (Fig. 1A) and endogenous cholinergic fiber restoration was observed following Aβ_1–42_ –induced neurotoxicity (Fig. 1D).

Decreasing levels of estradiol with menopause are associated with decreased cognitive function and progression of neurodegenerative disorders. Estren is a promising treatment alternative for hormone replacement therapy with beneficial effects for bone, vascular health and neurodegenerative conditions like Alzheimer’s disease without unwanted estrogenic side effects^23^,^24^,^28^,^29^,^30^,^45^,^52^. Our findings described in this study increase the understanding and translational value of esten treatment, particularly for neurodegenerative conditions. AD is the most common neurodegenerative disease. Neuroprotective therapies in late stages of AD are ineffective due to massive neuronal death, which precedes symptoms of dementia. However, new diagnostic tools are being developed and with early diagnosis of neurodegenerative conditions estren or other ANGELS might offer promising therapeutic options for treatment of AD patients with earlier, mild stages of the disease.

Here we demonstrated *in vivo* that the non-classical estradiol signaling pathway activator estren can effectively ameliorate the Aβ_1–42_-induced morphological and behavioral deficits in the brain. Our results clearly imply that estren directly acts on cholinergic MAPK/CREB intracellular signaling system via ERα to restore the BFC neurons against Aβ_1–42_ neurotoxicity. Further studies are required to comprehensively characterize the action of the estren, or find a more effective activator than estren of the non-classical estradiol signaling pathway; this may provide a basis for a future therapeutic approach to alleviate cholinergic loss in AD.

## Methods

### Aβ_1–42_ preparation

The Aβ_1–42_ is routinely produced as a recombinant protein fused with maltose binding protein (MBP) with a proteolytic cleavage site for Factor X protease between the two segments (Wilson C. MSc Thesis, University of Otago, 2007). This strategy utilises the solubilizing character of the maltose binding protein (product of the *MalE* gene) to ensure expression of soluble protein at high concentration in *Escherichia coli.* After expression of this recombinant fusion protein in bacteria, the product was purified on an amylose column to which the MBP segment of the protein binds. Following binding to amylose resin, the pure fusion protein was eluted from the resin with maltose, and concentrated by ammonium sulphate precipitation. The carrier MBP was then cleaved off the fusion protein by Factor X protease, and the released Aβ_1–42_ isolated and further purified by hydrophobic chromatography with 0–50% v/v acetonitrile/0.1% v/v TFA, using FPLC. The fractions containing pure Aβ_1–42_ were detected immunologically with an antibody against residues 17–24 of Aβ_1–42_ and lyophilized to remove solvent. Mass spectrometry was used to confirm the expected molecular ion for the desired product. Before the intra-cerebral microinjection of this product, we dissolved the prepared monomer in artificial cerebrospinal fluid (ACSF: 147 mM Na^+^, 3.5 mM K^+^, 2 mM Ca^2+^, 1 mM Mg^2+^, pH 7.3) and ‘aged’ the solution at RT for 48 h to facilitate the formation of toxic soluble aggregates, as documented by SDS/PAGE. The optimal incubation time for our preparations of Aβ_1–42_ to produce the highly toxic oligomers is 48–120 h.

### Animals

All experiments were approved and performed in accordance with the regulations of the ANZCCART and the University of Otago Animal Ethics Committee. All mice were bred and housed at the University of Otago, Hercus-Taieri Resource Unit. The animals were maintained under conditions of 12-h light/dark cycle (lights on at 0700 h) with food and water available *ad libitum* except for the behavioural experiments. All experiments were performed on adult (10–12 weeks old) female mice. Four mouse lines were used: C57BL/6J wild type control; ERα^loxP/loxP^ control (ERα^loxP/loxP^, data not shown); neuron- specific ERα^lox/loxp^knockout mice CamkIICre; ERα^loxP/loxP^ (nERαKO) and their wild type siblings (WT)^53^,^54^.

### *In vivo* experiments

#### Aβ_1–42_ and estrogenic injections

Mice of all mouse lines were anesthetized with Avertin (0.1 ml/10 g body weight) and bilaterally ovariectomized (OVX). Two weeks after the OVX they were anesthetized with isoflurane and they were mounted in a stereotaxic apparatus, and given 1 μl of aged Aβ_1–42_ diluted in ACSF slowly (0.1 μl/min) into the NBM of the right hemisphere or bilaterally for the behavioural studies. Aβ_1–42_ was injected at the stereotaxic coordinates relative to bregma at anteroposterior (−0.7 mm), mediolateral (−2 mm), and dorsoventral (−3.75 and −4.75 mm, 0.5 μl at both coordinates) from dura. Control injections were performed using 1 μl ACSF or 1 μl 300 μM scrambled Aβ_1–42_ (AnaSpec). Based on the results the 20 μM Aβ_1–42_ dose and the 12 d survival time were selected for all subsequent experiments, except for the signaling experiments, where the animals were sacrificed 30 min after estren treatment. Estren or E2 was administered subcutaneously with different concentrations (estren: 0.3, 3.3 33 ng/g (Straloids); E2: 33 ng/g (Sigma)) 1 h after intracerebral injection of Aβ_1–42_.

### *In vitro* experiments

The acute brain slice preparation for assessing ERK1/2 and CREB phosphorylation *in vitro* has been described previously^55^,^56^. Briefly, female C57BL/6 J wild type mice were decapitated 2 weeks following OVX, their brains rapidly removed and placed in oxygenated ACSF. Coronal 300 μm thick slices were cut on a vibratome and the slices pre-incubated at 30 °C for 30 min in oxygenated ACSF. Slices were transferred into ACSF containing 33.3 ng/g estren or vehicle (<0.01% ethyl alcohol) with or without 0.5 μM TTX and amino acid receptor blocker cocktail (10 μM 6-cyano-7-nitroquinoxaline-2,3-dione disodium salt (CNQX), 20 μM l-2-amino-5-phosphonopentanoic acid (AP5), 50 μM picrotoxin and 2 μM strychnine) for 30 min. The slices were fixed then in 4% PFA, and 30 μm-thick coronal sections cut on a freezing microtome. Dual-labeling fluorescence immunohistochemistry and analysis for ERK1/2, pERK1/2, CREB and pCREB were performed as described below.

### Immunohistochemistry

Free-floating peroxidase-based immunohistochemistry for choline acetyltransferase (ChAT) was undertaken as described previously^32^. Briefly, brain sections were incubated with primary antibodies recognizing ChAT (1:2000; Chemicon). This was followed by biotinylated donkey anti-goat IgGs (1:200; Jackson) and the avidin-biotin-HRP complex (1:200; Vector Elite ABC kit, Vector Laboratories) incubations. Labeling was then visualized with nickel-diaminobenzidine tetrahydrochloride (DAB) using glucose oxidase that resulted in a black precipitate within the labeled cells.

Free-floating dual-label fluorescence immunohistochemistry was performed to detect pERK1/2, ERK1/2, pCREB, CREB within ChAT neurons as described previously^56^,^57^,^58^ and Aβ_1–42_ in the NBM (Suppl Fig. 1). Briefly, brain sections were incubated with one of the primary antibodies recognizing pERK1/2, ERK1/2, pCREB or CREB (pERK1/2, 1:500, ERK1/2, 1:500, pCREB, 1:100, CREB, 1:1000; Cell Signaling Technologies; Aβ_1–42_, 1:500, Thermo Fisher Scientific) followed by incubation with chicken anti-rabbit Alexa Fluor 647 secondary antibody (1:500; Life Technologies). The sections were then processed further for ChAT immunolabeling (1:2000; Chemicon). This was followed by biotinylated donkey anti-goat Alexa Fluor 488 secondary antibody (1:200; Life technologies, USA) incubation. Sections were mounted on slides, air dried, and then coverslipped with VectaShield mounting medium (Vector Laboratories Inc).

Specificities of the primary antibodies have been tested and reported previously^37^,^57^,^59^,^60^,^61^. The omission of the primary antibodies resulted in complete absence of the immunoreactivity.

### Acetylcholine esterase (AChE) histochemistry

AChE histochemistry with silver nitrate intensification was performed to label and visualize cholinergic fibers in the cortex^32^,^62^. Brain sections were incubated in sodium acetate buffered (0.1 M; pH 6) acetylthiocoline-iodide (0.05%), sodium citrate (0.1 M), copper sulfate (0.03 M), and potassium ferricyanide (5 mM) solution. This was followed with ammonium sulfide (1%) and then silver nitrate (1%) incubation.

### Analysis of histological data

All measurements were performed by an investigator blind to the experimental groupings. Cholinergic cell body and fiber loss was analyzed as described with slight modification^32^,^63^,^64^. ChAT-positive cell bodies were counted in the NBM (plate 34 –35) on both brain sides according to the Paxinos and Franklin (2001) brain atlas^65^. Three sections starting from the bregma −1.2, with 120 μm inter-sectional distance from each animal was selected and analyzed for ChAT cell counting and 10 cortical sections for the AChE fiber density measurements (plate 28–40). Effects of Aβ_1–42_ lesion are expressed by forming percentage ratios of cell numbers and fiber density in the ipsi- and contralateral hemispheres except the behavioural studies where bilateral injections were delivered and the values are normalized to the naïve control group. Sections with ChAT and AChE labeling were examined under an Olympus BX51 microscope. Using a Cell-P Image Analysis software (Olympus, Japan) after background subtraction and gray scale threshold determination the surface area density of cortical AChE-positive fibers was measured.

Using the same anatomical areas as described above, three sections from NBM were selected from each animal, and the numbers of single ChAT-positive and double-labeled (ChAT + ERK1/2 or ChAT + pERK1/2; ChAT + CREB or ChAT + pCREB) neurons were determined using a Zeiss LSM 710 upright confocal laser-scanning microscope^55^. ERK1/2, pERK1/2, CREB and pCREB in ChAT-immunoreactive neurons are presented as the percentage of total number of ChAT-immunoreactive neurons in NBM. Note, using immunohistochemistry these proteins were successfully detected in the NBM by several research groups, including our own^14^,^57^,^58^.

### Behavioural experiments and analysis

After 12 days (D12) post bilateral 33 ng/g Aβ_1–42_ injection the following behavioural tests were carried out (started at 09.00 h).

For the single pellet skilled reaching task (performed on d12-d27) a three lane Plexiglas reaching apparatus (30 cm deep, 10 cm wide, and 30 cm high for each lane) was constructed to allow simultaneous recording of three animals^34^. Mice were fasted to 90% of their body weight and maintained at this level for the full 2-week testing period. Animals were habituated during the first day by placing them into the lanes for 15 min. Next day the sugar pellets (20 mg, Bio-Serv) were freely available on the lane floor within tongue reach as well as just outside the slot opening. Pellets were gradually removed from the floor until only the pellets just outside of slot remained. Pellets were gradually moved further away from the slot (approximately 1 cm maximal distance) to force the mice to use their paw and not their tongue to reach for pellets. All mice were weighed daily and fed approximately 2 g of food after each training period to maintain their body weight at 90%. From day 2 pellets were presented one at a time and reaches were recorded with a video-recorder. Each animal was presented with a total of 15 pellets during each 15 min test period for 14 d. The reach was scored if the mouse successfully brought the pellet back to its mouth and consumed it^34^. The single pellet skilled reaching task was performed at the middle of the dark cycle when the animal’s motivation to eat is very high.

The novel object recognition paradigm (d30-d31) was used to evaluate recognition memory. The test was performed at the start of the dark cycle (3 h into the dark cycle) when the activity levels of animals are high. Animals were allowed to explore a set of two identical objects for a 5 min period, afterwards the mice were returned to their cages. The next day (24 h later) the animals were presented with a similar set of objects in the same environment, where one object was novel to them; they were allowed to freely explore the objects again for a 5 min period. A discrimination preference, for a novel over a familiar object was calculated as follows: time near a new object less the time near the old object, divided by time near the new object plus the time near the old object^66^. The total distance travelled in the arena during the habituation period was used as a measure of exploration. No significant differences were found in the travelled distance between the different experimental groups. Animals were monitored and the videos analyzed with the TopScan (CleverSys. Inc., USA) system.

### Statistical analysis

Data in all experiments were expressed as mean ± SEM, except the survival curve data. Data are analysed by one-way ANOVA followed by Bonferroni’s or Tukey’s *post hoc* test (estren time and dose dependence study) with a value of p < 0.05 considered significant. Repeated measure and/or two-way ANOVA was used to analyze the behavioural data, the *in vitro* signaling and ERα KO data. P values of *post hoc* tests were adjusted using the Bonferroni test with a nominal significance level of 0.05. All statistical analysis were performed using Statistica 7.0 (StatSoft) and Prism (version 6; GraphPad Software).

## Additional Information

**How to cite this article**: Kwakowsky, A. *et al.* Treatment of beta amyloid 1–42 (Aβ_1–42_)-induced basal forebrain cholinergic damage by a non-classical estrogen signaling activator *in vivo. Sci. Rep.* **6**, 21101; doi: 10.1038/srep21101 (2016).

## Supplementary Material

Supplementary Information

## References

[b1] Huang Y. & Mucke L. Alzheimer mechanisms and therapeutic strategies. Cell 148, 1204–1222 (2012). 22424230 10.1016/j.cell.2012.02.040 PMC3319071

[b2] Moodley K. K. & Chan D. The hippocampus in neurodegenerative disease. Frontiers of neurology and neuroscience 34, 95–108 (2014). 24777134 10.1159/000356430

[b3] Brun A. & Englund E. Regional pattern of degeneration in Alzheimer’s disease: neuronal loss and histopathological grading. Histopathology 5, 549–564 (1981). 7286917 10.1111/j.1365-2559.1981.tb01818.x

[b4] Whitehouse P. J., Struble R. G., Clark A. W. & Price D. L. Alzheimer disease: plaques, tangles, and the basal forebrain. Annals of neurology 12, 494 (1982). 7181455 10.1002/ana.410120517

[b5] Mesulam M. M. Cholinergic circuitry of the human nucleus basalis and its fate in Alzheimer’s disease. The Journal of comparative neurology 521, 4124–4144 (2013). 23852922 10.1002/cne.23415 PMC4175400

[b6] Maccioni R. B., Munoz J. P. & Barbeito L. The molecular bases of Alzheimer’s disease and other neurodegenerative disorders. Archives of medical research 32, 367–381 (2001). 11578751 10.1016/s0188-4409(01)00316-2

[b7] Kruger L. & Mandelkow E. M. Tau neurotoxicity and rescue in animal models of human Tauopathies. Curr Opin Neurobiol 36, 52–58 (2015). 26431808 10.1016/j.conb.2015.09.004

[b8] Lesne S. E. *et al.* Brain amyloid-beta oligomers in ageing and Alzheimer’s disease. Brain : a journal of neurology 136, 1383–1398 (2013). 23576130 10.1093/brain/awt062 PMC3634198

[b9] De-Paula V. J., Radanovic M., Diniz B. S. & Forlenza O. V. Alzheimer’s disease. Sub-cellular biochemistry 65, 329–352 (2012). 23225010 10.1007/978-94-007-5416-4_14

[b10] Selkoe D. J. Alzheimer’s disease: genes, proteins, and therapy. Physiological reviews 81, 741–766 (2001). 11274343 10.1152/physrev.2001.81.2.741

[b11] Wollen K. A. Alzheimer’s disease: the pros and cons of pharmaceutical, nutritional, botanical, and stimulatory therapies, with a discussion of treatment strategies from the perspective of patients and practitioners. Alternative medicine review : a journal of clinical therapeutic 15, 223–244 (2010). 21155625

[b12] Lee S. J. & McEwen B. S. Neurotrophic and neuroprotective actions of estrogens and their therapeutic implications. Annual review of pharmacology and toxicology 41, 569–591 (2001). 10.1146/annurev.pharmtox.41.1.569 11264469

[b13] Aggarwal P. & Gibbs R. B. Estrogen replacement does not prevent the loss of choline acetyltransferase-positive cells in the basal forebrain following either neurochemical or mechanical lesions. Brain research 882, 75–85 (2000). 11056186 10.1016/s0006-8993(00)02832-8

[b14] Horvath K. M. *et al.* 17beta-estradiol enhances cortical cholinergic innervation and preserves synaptic density following excitotoxic lesions to the rat nucleus basalis magnocellularis. Neuroscience 110, 489–504 (2002). 11906788 10.1016/s0306-4522(01)00560-7

[b15] Abraham I. M., Koszegi Z., Tolod-Kemp E. & Szego E. M. Action of estrogen on survival of basal forebrain cholinergic neurons: promoting amelioration. Psychoneuroendocrinology 34 Suppl 1, S104–112 (2009). 19560872 10.1016/j.psyneuen.2009.05.024

[b16] Henderson V. W. Oestrogens and dementia. Novartis Found Symp 230, 254–265; discussion 265–273 (2000). 10965513 10.1002/0470870818.ch18

[b17] Wang C. N., Chi C. W., Lin Y. L., Chen C. F. & Shiao Y. J. The neuroprotective effects of phytoestrogens on amyloid beta protein-induced toxicity are mediated by abrogating the activation of caspase cascade in rat cortical neurons. J Biol Chem 276, 5287–5295 (2001). 11083861 10.1074/jbc.M006406200

[b18] Behl C. Oestrogen as a neuroprotective hormone. Nature reviews Neuroscience 3, 433–442 (2002). 12042878 10.1038/nrn846

[b19] Singh M., Sumien N., Kyser C. & Simpkins J. W. Estrogens and progesterone as neuroprotectants: what animal models teach us. Front Biosci 13, 1083–1089 (2008). 17981614 10.2741/2746 PMC2586167

[b20] Genazzani A. R., Pluchino N., Luisi S. & Luisi M. Estrogen, cognition and female ageing. Hum Reprod Update 13, 175–187 (2007). 17135285 10.1093/humupd/dml042

[b21] Luine V. N. Estradiol and cognitive function: past, present and future. Hormones and behavior 66, 602–618 (2014). 25205317 10.1016/j.yhbeh.2014.08.011 PMC4318702

[b22] Gurney E. P., Nachtigall M. J., Nachtigall L. E. & Naftolin F. The Women’s Health Initiative trial and related studies: 10 years later: a clinician’s view. The Journal of steroid biochemistry and molecular biology 142, 4–11 (2014). 24172877 10.1016/j.jsbmb.2013.10.009

[b23] Manson J. E. Current recommendations: what is the clinician to do? Fertility and sterility 101, 916–921 (2014). 24680650 10.1016/j.fertnstert.2014.02.043

[b24] Kwakowsky A., Koszegi Z., Cheong R. Y. & Abraham I. M. Neuroprotective effects of non-classical estrogen-like signaling activators: from mechanism to potential implications. CNS & neurological disorders drug targets 12, 1219–1225 (2013). 24040818

[b25] Nilsson S. *et al.* Mechanisms of estrogen action. Physiological reviews 81, 1535–1565 (2001). 11581496 10.1152/physrev.2001.81.4.1535

[b26] Vasudevan N. & Pfaff D. W. Non-genomic actions of estrogens and their interaction with genomic actions in the brain. Frontiers in neuroendocrinology 29, 238–257 (2008). 18083219 10.1016/j.yfrne.2007.08.003

[b27] Micevych P. & Dominguez R. Membrane estradiol signaling in the brain. Frontiers in neuroendocrinology 30, 315–327 (2009). 19416735 10.1016/j.yfrne.2009.04.011 PMC2720427

[b28] Zhao L., Chen S., Ming Wang J. & Brinton R. D. 17beta-estradiol induces Ca2+ influx, dendritic and nuclear Ca2+ rise and subsequent cyclic AMP response element-binding protein activation in hippocampal neurons: a potential initiation mechanism for estrogen neurotrophism. Neuroscience 132, 299–311 (2005). 15802184 10.1016/j.neuroscience.2004.11.054

[b29] Dominguez R., Jalali C. & de Lacalle S. Morphological effects of estrogen on cholinergic neurons *in vitro* involves activation of extracellular signal-regulated kinases. The Journal of neuroscience : the official journal of the Society for Neuroscience 24, 982–990 (2004). 14749443 10.1523/JNEUROSCI.2586-03.2004 PMC3182120

[b30] Lee S. J. *et al.* Estrogen induces phosphorylation of cyclic AMP response element binding (pCREB) in primary hippocampal cells in a time-dependent manner. Neuroscience 124, 549–560 (2004). 14980726 10.1016/j.neuroscience.2003.11.035

[b31] Marin R. *et al.* Estradiol prevents amyloid-beta peptide-induced cell death in a cholinergic cell line via modulation of a classical estrogen receptor. Neuroscience 121, 917–926 (2003). 14580942 10.1016/s0306-4522(03)00464-0

[b32] Koszegi Z., Szego E. M., Cheong R. Y., Tolod-Kemp E. & Abraham I. M. Postlesion estradiol treatment increases cortical cholinergic innervations via estrogen receptor-alpha dependent nonclassical estrogen signaling *in vivo*. Endocrinology 152, 3471–3482 (2011). 21791565 10.1210/en.2011-1017

[b33] Kousteni S. *et al.* Reversal of bone loss in mice by nongenotropic signaling of sex steroids. Science 298, 843–846 (2002). 12399595 10.1126/science.1074935

[b34] Conner J. M., Culberson A., Packowski C., Chiba A. A. & Tuszynski M. H. Lesions of the Basal forebrain cholinergic system impair task acquisition and abolish cortical plasticity associated with motor skill learning. Neuron 38, 819–829 (2003). 12797965 10.1016/s0896-6273(03)00288-5

[b35] Cordey M., Gundimeda U., Gopalakrishna R. & Pike C. J. The synthetic estrogen 4-estren-3 alpha,17 beta-diol (estren) induces estrogen-like neuroprotection. Neurobiology of disease 19, 331–339 (2005). 15837589 10.1016/j.nbd.2005.01.011

[b36] Falkenstein E., Tillmann H. C., Christ M., Feuring M. & Wehling M. Multiple actions of steroid hormones–a focus on rapid, nongenomic effects. Pharmacological reviews 52, 513–556 (2000). 11121509

[b37] Kalesnykas G., Roschier U., Puolivali J., Wang J. & Miettinen R. The effect of aging on the subcellular distribution of estrogen receptor-alpha in the cholinergic neurons of transgenic and wild-type mice. The European journal of neuroscience 21, 1437–1442 (2005). 15813954 10.1111/j.1460-9568.2005.03953.x

[b38] Forny-Germano L. *et al.* Alzheimer’s disease-like pathology induced by amyloid-beta oligomers in nonhuman primates. The Journal of neuroscience : the official journal of the Society for Neuroscience 34, 13629–13643 (2014). 25297091 10.1523/JNEUROSCI.1353-14.2014 PMC6608380

[b39] Giovannelli L., Casamenti F., Scali C., Bartolini L. & Pepeu G. Differential effects of amyloid peptides beta-(1–40) and beta-(25–35) injections into the rat nucleus basalis. Neuroscience 66, 781–792 (1995). 7651609 10.1016/0306-4522(94)00610-h

[b40] Harkany T. *et al.* Beta-amyloid(Phe(SO3H)24)25–35 in rat nucleus basalis induces behavioral dysfunctions, impairs learning and memory and disrupts cortical cholinergic innervation. Behavioural brain research 90, 133–145 (1998). 9580273 10.1016/s0166-4328(97)00091-0

[b41] Abraham I. *et al.* Chronic corticosterone administration dose-dependently modulates Abeta(1–42)- and NMDA-induced neurodegeneration in rat magnocellular nucleus basalis. Journal of neuroendocrinology 12, 486–494 (2000). 10844576 10.1046/j.1365-2826.2000.00475.x

[b42] Gotz J. & Ittner L. M. Animal models of Alzheimer’s disease and frontotemporal dementia. Nature reviews Neuroscience 9, 532–544 (2008). 18568014 10.1038/nrn2420

[b43] Wirths O. & Bayer T. A. Neuron loss in transgenic mouse models of Alzheimer’s disease. International journal of Alzheimer’s disease 2010, 1–6 (2010). 10.4061/2010/723782 PMC2943100 20871861

[b44] Wirths O., Dins A. & Bayer T. A. AbetaPP accumulation and/or intraneuronal amyloid-beta accumulation? The 3xTg-AD mouse model revisited. Journal of Alzheimer’s disease : JAD 28, 897–904 (2012). 22112547 10.3233/JAD-2011-111529

[b45] Singer C. A., Figueroa-Masot X. A., Batchelor R. H. & Dorsa D. M. The mitogen-activated protein kinase pathway mediates estrogen neuroprotection after glutamate toxicity in primary cortical neurons. The Journal of neuroscience : the official journal of the Society for Neuroscience 19, 2455–2463 (1999). 10087060 10.1523/JNEUROSCI.19-07-02455.1999 PMC6786088

[b46] Kim J. S. *et al.* Enhancement of rat hippocampal long-term potentiation by 17 beta-estradiol involves mitogen-activated protein kinase-dependent and -independent components. Neuroscience letters 332, 65–69 (2002). 12377386 10.1016/s0304-3940(02)00902-3

[b47] Guerra B., Diaz M., Alonso R. & Marin R. Plasma membrane oestrogen receptor mediates neuroprotection against beta-amyloid toxicity through activation of Raf-1/MEK/ERK cascade in septal-derived cholinergic SN56 cells. Journal of neurochemistry 91, 99–109 (2004). 15379891 10.1111/j.1471-4159.2004.02695.x

[b48] Carlstrom L., Ke Z. J., Unnerstall J. R., Cohen R. S. & Pandey S. C. Estrogen modulation of the cyclic AMP response element-binding protein pathway. Effects of long-term and acute treatments. Neuroendocrinology 74, 227–243 (2001). 11598379 10.1159/000054690

[b49] Bora S. H., Liu Z., Kecojevic A., Merchenthaler I. & Koliatsos V. E. Direct, complex effects of estrogens on basal forebrain cholinergic neurons. Experimental neurology 194, 506–522 (2005). 15893308 10.1016/j.expneurol.2005.03.015

[b50] Dubal D. B. *et al.* Estrogen receptor alpha, not beta, is a critical link in estradiol-mediated protection against brain injury. Proceedings of the National Academy of Sciences of the United States of America 98, 1952–1957 (2001). 11172057 10.1073/pnas.041483198 PMC29363

[b51] Milne M. R., Haug C. A., Abraham I. M. & Kwakowsky A. Estradiol modulation of neurotrophin receptor expression in female mouse basal forebrain cholinergic neurons *in vivo*. Endocrinology 156, 613–626 (2015). 25415243 10.1210/en.2014-1669

[b52] Gibbs R. B. Estrogen therapy and cognition: a review of the cholinergic hypothesis. Endocrine reviews 31, 224–253 (2010). 20019127 10.1210/er.2009-0036 PMC2852210

[b53] Wintermantel T. M. *et al.* Definition of estrogen receptor pathway critical for estrogen positive feedback to gonadotropin-releasing hormone neurons and fertility. Neuron 52, 271–280 (2006). 17046690 10.1016/j.neuron.2006.07.023 PMC6116893

[b54] Casanova E. *et al.* A CamKIIalpha iCre BAC allows brain-specific gene inactivation. Genesis 31, 37–42 (2001). 11668676 10.1002/gene.1078

[b55] Cheong R. Y. *et al.* Estradiol acts directly and indirectly on multiple signaling pathways to phosphorylate cAMP-response element binding protein in GnRH neurons. Endocrinology 153, 3792–3803 (2012). 22719057 10.1210/en.2012-1232

[b56] Abraham I. M., Han S. K., Todman M. G., Korach K. S. & Herbison A. E. Estrogen receptor beta mediates rapid estrogen actions on gonadotropin-releasing hormone neurons *in vivo*. The Journal of neuroscience : the official journal of the Society for Neuroscience 23, 5771–5777 (2003). 12843281 10.1523/JNEUROSCI.23-13-05771.2003 PMC6741236

[b57] Barabas K. *et al.* Sex differences in oestrogen-induced p44/42 MAPK phosphorylation in the mouse brain *in vivo*. Journal of neuroendocrinology 18, 621–628 (2006). 16867183 10.1111/j.1365-2826.2006.01447.x

[b58] Szego E. M. *et al.* Estrogen induces estrogen receptor alpha-dependent cAMP response element-binding protein phosphorylation via mitogen activated protein kinase pathway in basal forebrain cholinergic neurons *in vivo*. The Journal of neuroscience : the official journal of the Society for Neuroscience 26, 4104–4110 (2006). 16611827 10.1523/JNEUROSCI.0222-06.2006 PMC6673875

[b59] Yeo T. T. *et al.* Absence of p75NTR causes increased basal forebrain cholinergic neuron size, choline acetyltransferase activity, and target innervation. The Journal of neuroscience : the official journal of the Society for Neuroscience 17, 7594–7605 (1997). 9315882 10.1523/JNEUROSCI.17-20-07594.1997 PMC6793892

[b60] McNulty S., Schurov I. L., Sloper P. J. & Hastings M. H. Stimuli which entrain the circadian clock of the neonatal Syrian hamster *in vivo* regulate the phosphorylation of the transcription factor CREB in the suprachiasmatic nucleus *in vitro*. The European journal of neuroscience 10, 1063–1072 (1998). 9753174 10.1046/j.1460-9568.1998.00114.x

[b61] von Gall C. *et al.* CREB in the mouse SCN: a molecular interface coding the phase-adjusting stimuli light, glutamate, PACAP, and melatonin for clockwork access. The Journal of neuroscience : the official journal of the Society for Neuroscience 18, 10389–10397 (1998). 9852576 10.1523/JNEUROSCI.18-24-10389.1998 PMC6793329

[b62] Hedreen J. C., Bacon S. J. & Price D. L. A modified histochemical technique to visualize acetylcholinesterase-containing axons. The journal of histochemistry and cytochemistry : official journal of the Histochemistry Society 33, 134–140 (1985). 2578498 10.1177/33.2.2578498

[b63] Horvath K. M. *et al.* Postnatal treatment with ACTH-(4–9) analog ORG 2766 attenuates N-methyl-D-aspartate-induced excitotoxicity in rat nucleus basalis in adulthood. European journal of pharmacology 405, 33–42 (2000). 11033312 10.1016/s0014-2999(00)00539-2

[b64] Harkany T. *et al.* Short-term consequences of N-methyl-D-aspartate excitotoxicity in rat magnocellular nucleus basalis: effects on *in vivo* labelling of cholinergic neurons. Neuroscience 108, 611–627 (2001). 11738498 10.1016/s0306-4522(01)00443-2

[b65] Paxinos G. & Franklin K. The mouse brain in stereotaxic coordinates . 2nd ed. (San Diego: Academic Press (2000).

[b66] Dix S. L. & Aggleton J. P. Extending the spontaneous preference test of recognition: evidence of object-location and object-context recognition. Behavioural brain research 99, 191–200 (1999). 10512585 10.1016/s0166-4328(98)00079-5

