# Supplementary material for: Treatment of beta amyloid 1–42 (Aβ1–42)-induced basal forebrain cholinergic damage by a non-classical estrogen signaling activator in vivo
Source: Sci Rep. 2016 Feb 16;6:21101. doi: 10.1038/srep21101 (PMC4754683; doi:10.1038/srep21101)
Supplement: Supplementary Information [file srep21101-s1.pdf]

Treatment of beta amyloid 1-42 (Aβ_{1-42})-induced basal forebrain cholinergic damage by a non-classical estrogen signaling activator *in vivo*

Andrea Kwakowsky, Kyoko Potapov, SooHyun Kim, Katie Peppercorn,
Warren P. Tate and István M. Ábrahám

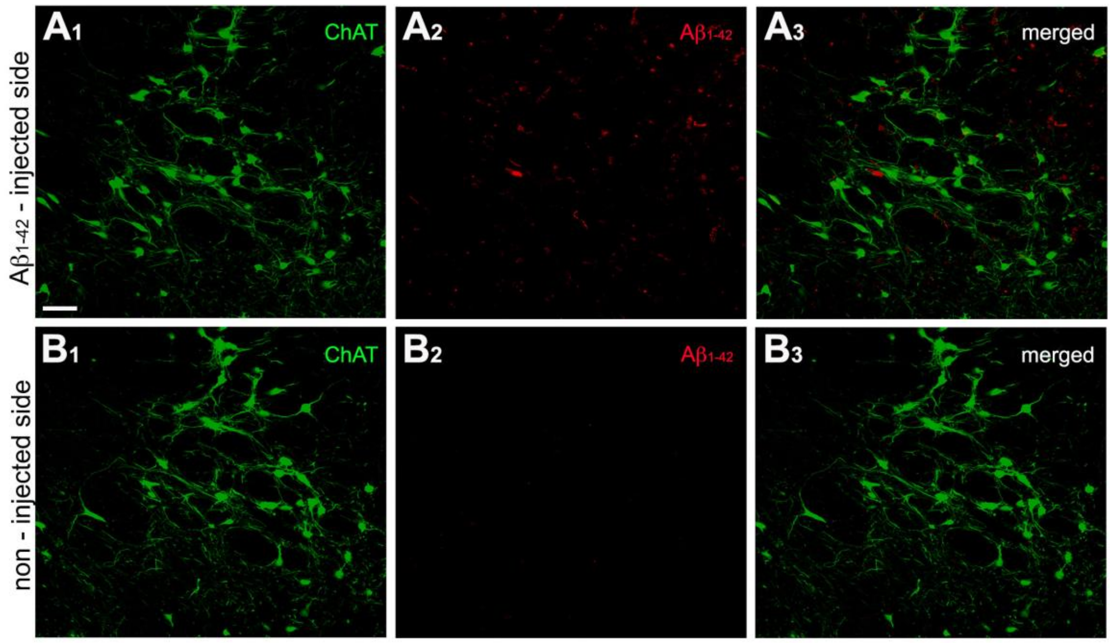

Photomicrographs demonstrate ChAT positive cell bodies in the NBM and $A\beta_{1\text{-}42}$ immunolabeling at the non-injected control brain side (A) and $A\beta_{1\text{-}42}$ injected side (B) after 90 min of $A\beta_{1\text{-}42}$ injection. Scale bar, 40 μm.
